# *Rickettsia raoultii* in *Dermacentor reticulatus* Ticks, Chernobyl Exclusion Zone, Ukraine, 2010

**DOI:** 10.3201/eid2212.160678

**Published:** 2016-12

**Authors:** Grzegorz Karbowiak, Kateryna Slivinska, Tomasz Chmielewski, Kamila Barszcz, Stanisława Tylewska-Wierzbanowska, Joanna Werszko, Tomasz Szewczyk, Piotr Wróblewski

**Affiliations:** W. Stefański Institute of Parasitology, Polish Academy of Sciences, Warsaw, Poland (G. Karbowiak, J. Werszko, T. Szewczyk, P. Wróblewski);; I.I. Schmalhausen Institute of Zoology, National Academy of Sciences of Ukraine, Kiev, Ukraine (K. Slivinska);; National Institute of Public Health–National Institute of Hygiene, Warsaw (T. Chmielewski, K. Barszcz, S. Tylewska-Wierzbanowska)

**Keywords:** *Rickettsia raoultii*, *Dermacentor reticulatus*, Chernobyl Exclusion Zone, Ukraine, vector-borne infections, ticks, rickettsia, bacteria

**To the Editor:** The Chernobyl Exclusion Zone (CEZ) surrounds the center of the 1986 Chernobyl nuclear power plant disaster. Preliminary study shows predominance of *Dermacentor reticulatus* ticks in the CEZ; ticks of other species, such as *Ixodes ricinus*, are surprisingly rare, even in habitats where they should be relatively common (*1*). A few reports document presence of *Ix. trianguliceps* ticks (*2**,**3*). Prevalence of pathogens (*Anaplasma phagocytophilum*, *Borrelia burgdorferi* s.l., *Babesia* spp.) in these ticks is higher in the CEZ than in other regions (*3**,**4*). One pathogen transmitted by *Dermacentor* spp. ticks is *Rickettsia raoultii*, which has been isolated from species of *Dermacentor* ticks found in Asia (*5**,**6*) and since 1999 has also been detected in Europe. 

In our study, *D. reticulatus* ticks were collected by use of the flagging method (*1*) in the CEZ in September 2010. Ticks were collected from areas where they were known to occur, around the former villages of Korohod (51°16′02′′N; 30°01′04′′E) and Cherevach (51°12′44′′N; 30°07′45′′E) and around Chernobyl city (51°17′04′′N; 30°13′25′′E). The habitats investigated included open areas and the remnants of farmlands. A total of 201 *D. reticulatus* ticks, 87 males and 114 females, were collected and investigated (Table). 

**Table:** Prevalence of *Rickettsia raoultii* infection among *Dermacentor reticulatus* ticks, Chernobyl Exclusion Zone, Ukraine, 2010

Area of tick collection	No. ticks infected/no. ticks examined (prevalence, %)		
	Male	Female	Total
Korohod	40/48 (83.33)	40/60 (66.66)	80/108 (74.07)
Cherevach	21/28 (75.00)	32/46 (69.56)	53/74 (71.62)
Chernobyl	9/11 (81.82)	4/8 (50.00)	13/19 (68.42)
All 3 areas	70/87 (80.46)	76/114 (66.66)	146/201 (72.64)

DNA was extracted by use of the ammonium hydroxide method (*7*). Isolated DNA was examined for the presence of the *Rickettsia* sp. citrate synthase gene (*gltA*) by use of PCR with *Rp*CS.409d and *Rp*CS.1258n primers (*8*). Positive amplicons were sequenced, and sequences were edited by using AutoAssembler software (Applied Biosystems, Foster City, CA, USA) and compared with GenBank entries by using blastn version 2.2.13 (http://www.ncbi.nlm.nih.gov/blast/download.shtml). All obtained sequences were submitted to GenBank (accession nos. KX056493 and KX056494).

Infection with *Rickettsia* spp. was detected in 72.64% of ticks (Table). A higher proportion of males (80.46%) than females (66.66%) were infected. Sequence analysis showed 100% identity with *R. raoultii* isolated from *D. marginatus* ticks from China (GenBank accession nos. KU171018.1 and KT261764.1) and *D. reticulatus* ticks from Poland (KT277489) and Hungary (LC060714.1). In the CEZ, the predominant tick species is *D. reticulatus*; no *D. marginatus* ticks have been found in the CEZ (*1*). Thus, in this area, the *R. raoultii* vector is *D. reticulatus* ticks. 

The prevalence of *R. raoultii* infection among *D. reticulatus* ticks (68.42%–74.07%) is significantly higher in the CEZ than in other regions. A previous study found prevalence of *A. phagocytophilum* infection in the CEZ to be high, mainly associated with *Ixodes* ticks (*9*) and rarely associated with *D. reticulatus* ticks. The prevalence of *Babesia canis* infection, also vectored by this tick, was within the usual range (*4*). The reason for prevalence of at least 2 vectored pathogens being higher in *D. reticulatus* ticks in the CEZ than in other region is not known and needs more study. The prevalence of these pathogens among mammals that inhabit the CEZ is also not known; the influence of radiation on pathogen level has not been studied. The nucleotide sequences of *R. raoultii* detected in ticks in the CEZ are identical to sequences originating from other regions and deposited in GenBank; the sequences of *A. phagocytophilum* and *B. canis* from the CEZ were also similar to those described elsewhere (*4*). If the reason for the higher *R. raoultii* infection prevalence is radiation, then radiation also influences the ticks—some morphologic abnormalities have been noted on *D. reticulatus* ticks collected from the CEZ (*10*). 

This study confirms presence of *R. raoultii* in *D. reticulatus* ticks in the CEZ. The structure of zoonotic foci in the CEZ seems to differ from that in other regions. Confirmation of this hypothesis needs follow-up study of tickborne pathogens in wild mammals that might serve as a source of infection for ticks in the CEZ. 
